# Mr. Hennen, on the Use of Sponge in Ophthalmia

**Published:** 1804-09-01

**Authors:** John Hennen

**Affiliations:** Surgeon, 3d Division of Light Infantry. Strabane


### *To the Editors of the Medical and Physical Journal.*

GENTLEMEN,

THE good effects which I have frequently experienced from the external application of *Sponge,* lead me to submit to you the following observations on its efficacy as a dressing in many cases, in which I believe it is not at present in general use.

The first I shall mention, and indeed one in which I have never failed in giving the most marked relief, is Ophthalmia. The usual mode of applying refrigerant and astringent applications to an inflamed eye, is either by means of linen compresses or bread cataplasms, soaked in a saturnine solution. These are in many cases ineffectual, and in all inelegant, modes of dressing; the linen very shortly becomes exceedingly stiff, and the coarser particles of the lead being precipitated, give a constant and violent stimulus to the organ. Bread, although free in a great measure from the latter fault, produces so great an accumulation of heat in the diseased part, that it is rendered hard in a very short time; and if long applied, is reduced to a half-baked state. If this is attempted to be obviated by frequent renewal, the admission of light, in a great measure, counteracts the design of the application, and at night is impracticable. To avoid those inconveniencies, I am in the habit of applying a bandage composed of two circular pieces of fine sponge, extremely well beaten and washed, so as to remove any extraneous matter which might adhere to them, and then sewed to a bit of tape, so as to compleatly cover both eyes, and tie behind the head. If one eye only is diseased, the piece of sponge corresponding with it, is soaked in the application intended to be used, while the other excludes the light from the sound eye; an object of material consequence in every affection of those delicate organs, whose motions sympathize so strongly. By this means the remedy is kept constantly applied to the diseased part, the dressings never grow stiff, the stimulus of light is effectually excluded, and a greater and more uniform degree of cold is produced than can possibly be done by any other means, as a larger and freer surface is exposed to evaporation; added to which, the supply is constant, an object of great comfort to the patient, particularly at night, as he needs only to press the sponge and produce the desired quantity. I have at present a case under under my care, of the most violent ophthalmia I ever saw, and differing only from that which affected our troops in the late expedition to Egypt, in not being contagious; it had resisted every topical application to the eye itself. Blisters to the temples and behind the ears, purging with repeated doses of calomel, and even scarifications, had produced but a temporary relief. The application of the sponge however, immersed in a solution of vitriolated zinc, in twelve hours produced so favourable a change, as to do away the fears of suppuration taking place; and in the course of the ensuing day a rapid progress was made towards a cure; he is now in a state of convalescence. The sponge dressing is eminently useful in inflammation of the testicle; the usual application of a cataplasm impregnated with saturnine solution is not only extremely filthy, but from the great heat produced becomes very soon stiff, while a small bag of oiled silk or varnished linen, lined with a layer of prepared sponge, keeps the part constantly cool, moist, and immersed in the remedy, and acts as an excellent suspensory bandage. If the disease is produced from a gonorrhœa too suddenly stopped, a case of the same material with a bit of sponge soaked in warm oil, sewed in the extremity of it, will keep the glans constantly moist, solicit a renewal of the discharge, and together with the injection of warm oil, ensure a speedy termination of the complaint. And indeed, in every case of gonorrhœa, where cleanliness is indispensable and concealment frequently necessary, a case for the penis of this kind, with the sponge soaked either in an astringent or emollient application, as may be judged necessary, will be found extremely useful. In inflammatory affections of the extremities, particularly that species of paronychia, which runs so rapidly to a termination, as frequently to render amputation necessary, this dressing will be found excellent when impregnated with a very strong astringent. In almost every case of external inflammation, the medicated sponge is superior to any other application; and where, from the shape or situation of the part, it can be applied without any external covering (as the eye or knee). The rapidity of evaporation and consequent decrease of temperature will considerably augment the effect. When emollient applications and an increase of heat are indicated, both those intentions will be fully answered by applying the case lined with sponge immersed in warm oil. The relaxation of the surface produced is very rapid and effectual; in many cases so much so, as to produce a profuse perspiration perspiration on the part. A particular friend, a patient of mine, who has been long a martyr to gout, is constantly furnished with an apparatus of this nature, which in him uniformly produces a great degree of perspiration and consequent relief of the fit.

My opportunities of trying this mode of dressing have been principally confined to inflammatory cases; and the good effects produced from it, in them, have been so obvious as to induce me to give a decided preference to it, where it can possibly be procured.

I am, &c.

*Strabane,*

*August* 4, 1804.

JOHN HENNEN, Surgeon,
3d Division of Light Infantry.

